# Crust-mantle decoupling beneath Afar revealed by Rayleigh-wave tomography

**DOI:** 10.1038/s41598-022-20890-5

**Published:** 2022-10-11

**Authors:** Utpal Kumar, Cédric P. Legendre

**Affiliations:** 1 Berkeley Seismological Laboratory, University of California, Berkeley, 307 McCone Hall #4760, Berkeley, CA 94720-4760 USA; 2 Institute of Geophysics, Czech Academy of Science, Boční II/1401, 14131 Praha, Czech Republic

**Keywords:** Geophysics, Solid Earth sciences, Seismology

## Abstract

The Afar triple junction accustoms the diverging plate dynamics between the Arabian, Nubian, and Somalian plates along the Red Sea, Gulf of Aden, and East African rifts. The average anisotropy obtained from shear-wave splitting measurements agrees with the surface motion recovered by geodetic analyses. However, the vertical layering of anisotropy in this region is yet to be accurately determined. Here, we use earthquake seismic data to map Rayleigh-wave azimuthal anisotropy in the crust and lithospheric mantle beneath the East African Rift System. Our results suggest that a layering of anisotropy is present around the East African Rift System. At shorter periods that sample the crust, rift-parallel anisotropy is present in the vicinity of the rift, but in the central part of the rift, rift-normal anisotropy is found. At longer periods, sampling the lithospheric mantle, the anisotropic pattern is quite different. These observations suggest that the crust and lithospheric mantle are mechanically decoupled beneath the environs of the East African Rift System. Similarly, these results suggest complex dynamics within the crust and lithosphere in the region of the Afar triple junction.

## Introduction

**Figure 1:**
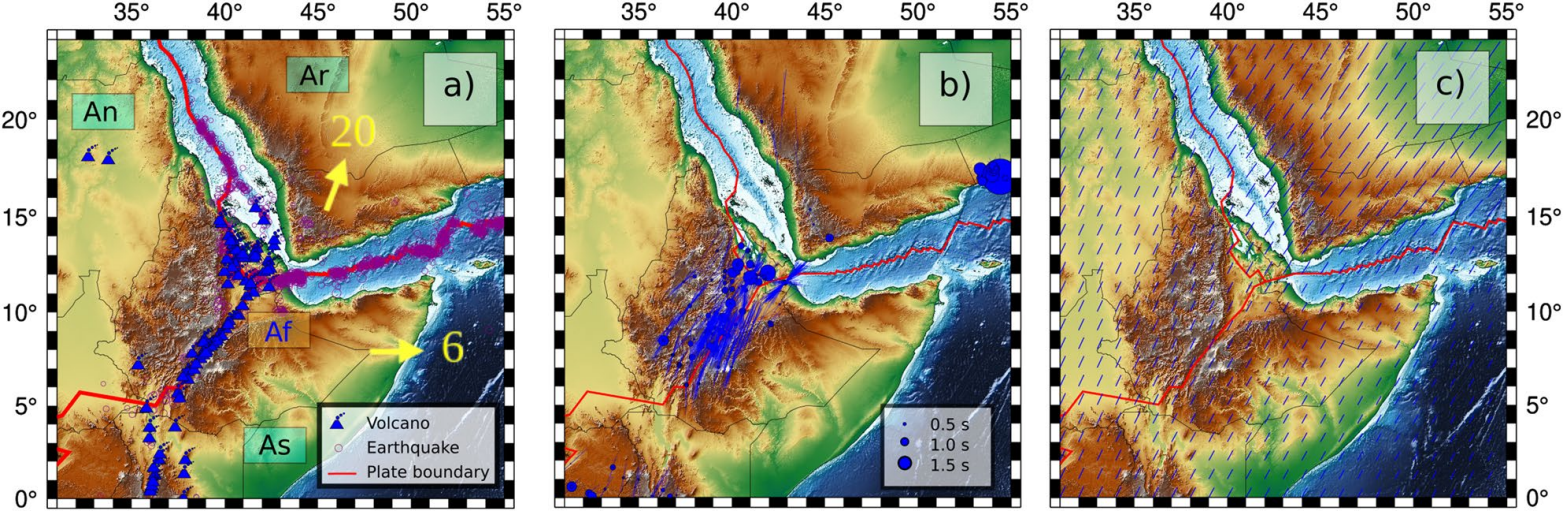
(**a**) Regional tectonic map of Afar region. Plate boundaries^1^ are framed in red. The yellow arrows indicate the Absolute Plate Motion (in mm/year) with a European reference^49^. The location of the Holocene volcanoes^43^ are highlighted with blue volcanoes shape. The location of the epicenters of earthquakes^50^ is indicated with purple circles (Events depth displayed in Fig. S1). The black text framed in green provides the names of the different plates^1^: An, African (Nubian) plate; As, African (Somalian) plate; Ar, Arabian plate. The blue text framed in brown (Af) indicates the location of the Afar triple junction. (**b**) Shear-wave splitting measurements^29,30^. (**c**) GPS velocity field in a Eurasia-fixed reference frame^23^. Individual figure panels were generated with the Generic Mapping Tool (6.3.0)^51^ and PyGMT (0.7.0)^52^. Individual figure panels were combined using Inkscape (1.2.1)^53^.

The Afar rift, located at the northern tip of the East African Rift System^1^, is a region with high crustal seismicity and active volcanoes (Figs. 1a and S1), because of the last stage of continental rifting and the early stage of seafloor spreading^2,3^.

The Afar hotspot is located at the triple junction between the Red Sea rift, the Gulf of Aden, and the Ethiopian rift zone. It is rising at the triple junction, mingling the three branches of the East African Rift System^4–6^. The East African Rift System is a divergent tectonic plate boundary splitting the African Plate into two: the Nubian and Somalian plates^1^. The Red Sea rift separates the Arabian and Nubian-African plates, whereas the Gulf of Aden separates the Arabian and Somalian-African plates^1^.

The influence of the mantle upwelling of the Afar hotspot on the East African Rift System has been the topic of numerous studies^4,7^. Seismic imaging allows to extract important details on the structure and deformation to map the crustal and mantle structures, hence providing information on the regional tectonic system. In addition, seismic anisotropic studies can yield information related to the geodynamical evolution of the sampled material, which is a key factor in understanding the geodynamical mechanisms of the region^8,9^.

Recent seismological studies of the region have shown remarkably low seismic velocity in the central Afar^10–12^. The low velocities, not only limited to the center of the rift, are mainly associated with active tectonic and magmatic activities. Exceptionally negative seismic velocity anomalies are found in the crust and upper mantle, up to 5–15% slower than the PREM^13^. Such anomalies can be only explained by the combined effects of temperature and partial melt^14,15^. The Afar hotspot has been suspected to have a very deep origin, likely in the lower mantle and is associated with the African Superplume^16^, with very strong velocity variations in the mantle transition zone and upper mantle^17,18^.

Lateral and vertical variations in the seismic anisotropy can provide additional constraints on the tectonic evolution of the crust and lithospheric material of a selected region^19,20^. Numerous studies focusing on shear-wave splitting measurements^7,21,22^ display a very homogeneous pattern of anisotropy (Fig. 1b), which is consistent with the surface velocity vectors^23^ (Fig. 1c) obtained from geodetic measurements. The presence of radial anisotropy^24^ suggests an intrinsically layered crust, with an accumulation of sills in the upper to the mid crust, both on and off-rift.

**Figure 2:**
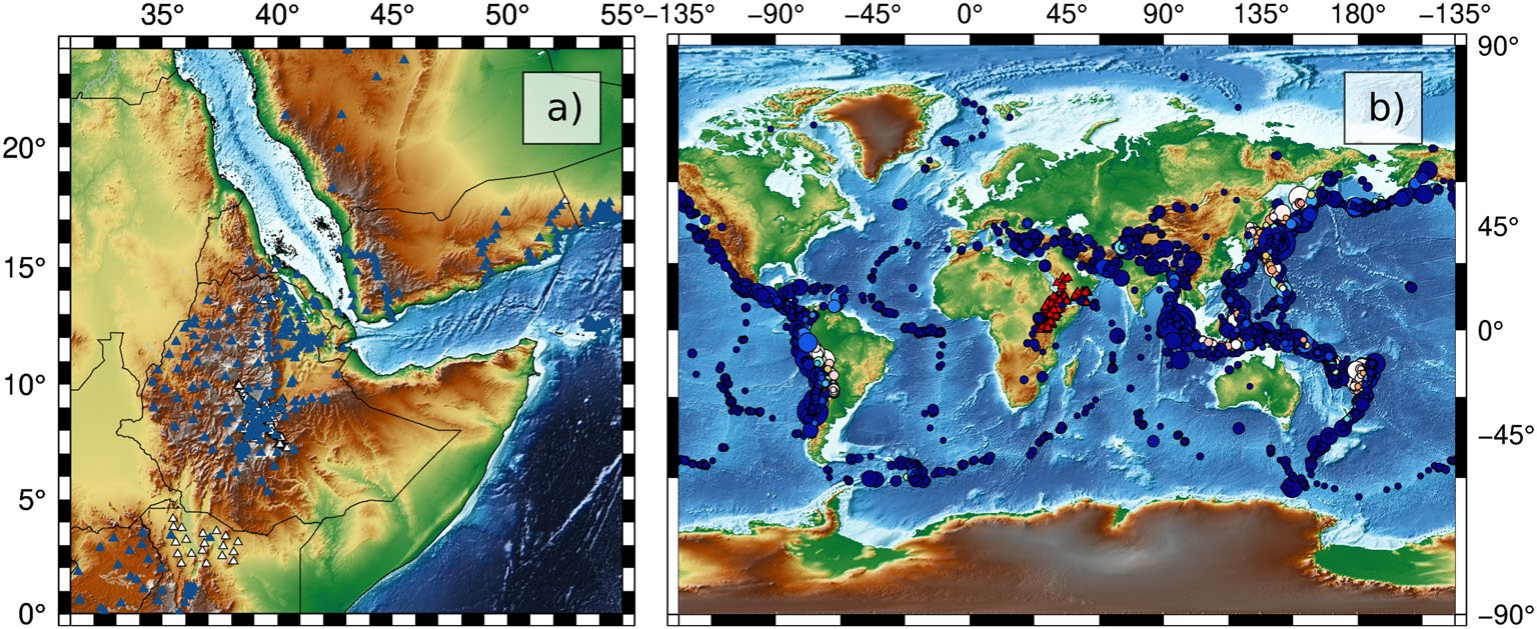
Map of the 361 available seismic stations (**a**) and 4,137 selected regional and teleseismic earthquakes (**b**). Individual figure panels were generated with the Generic Mapping Tool (6.3.0)^51^ and PyGMT (0.7.0)^52^. Individual figure panels were combined using Inkscape (1.2.1)^53^.

In this study, we used the vertical component of open-access seismic records in the region to build tomographic maps of the fundamental-mode Rayleigh-wave phase velocity for the Afar triple junction. First, we obtain all seismic data available in the region. A total of 361 seismic stations (Fig. 2a) recording 4137 regional and teleseismic earthquakes (Fig. 2b) between the years 1990–2021 and magnitude (M$_W$) > 6.0 were used^25^. For each pair of station, we selected all events with an epicentral distance at least five times larger than the inter-station distance. Then, we kept only the events for which the difference in back-azimuth between the event and both stations was smaller than ± 5$^{\circ }$.

**Figure 3:**
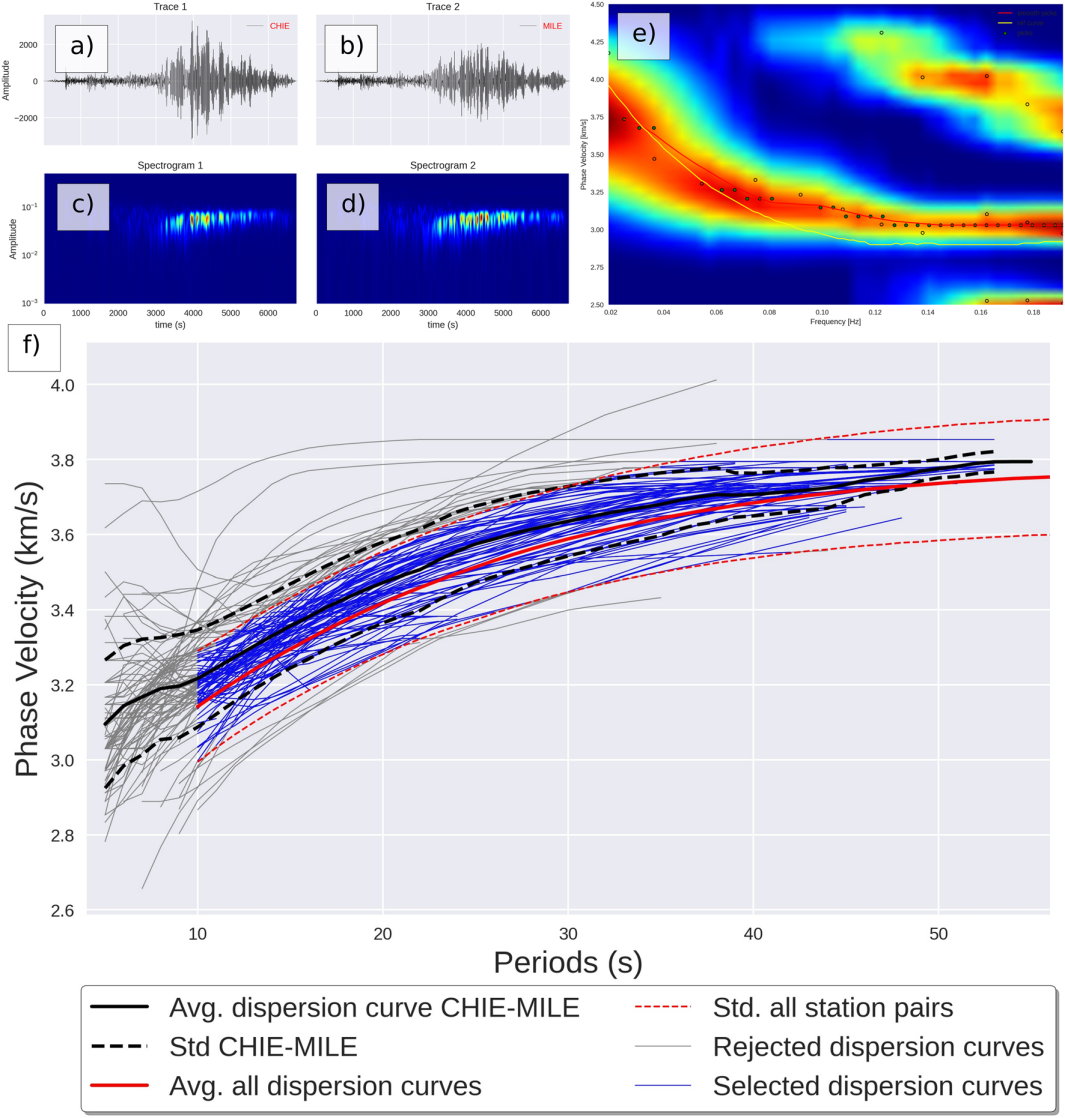
Example seismic traces of an event (M$_W$ = 6.8) on 2007-09-10 01:49:14 (3.0$^\circ$N, − 77.9$^\circ$E, 29 km depth), recorded at two stations ZE.CHIE (**a**) and ZE.MILE (**b**) and respective spectrogram (**c** & **d**). Cross-correlation function and phase picking. The dispersion curve was automatically selected with successful pick (green circles) or unsuccessful selection (empty circles) using the spectrum’s zero crossing (**e**). Summary of all fragments of dispersion curves picked for this station pair and average dispersion curve (**f**).

For each selected event satisfying the magnitude / distance / back-azimuth criteria (Fig. S2), the seismic records (Fig. 3a,b) were first transferred in the frequency domain (Fig. 3c,d), cross-correlated and the phase velocities were computed^26^ (Fig. 3e). A total of 68,420 dispersion curves were measured (Fig. S3) to constrain 3,578 path-average dispersion curves^26,27^. For each individual dispersion curve, statistical analysis was performed. Each individual fragment was compared with the average of all dispersion curves as well as with the path-specific average. Fragments displaying excessive deviation (over one standard deviation) were discarded, and the pair specific averaged dispersion curve was recomputed (Fig. 3f). Path-specific dispersion curves constrained by fewer than ten measurements were discarded.

**Figure 4:**
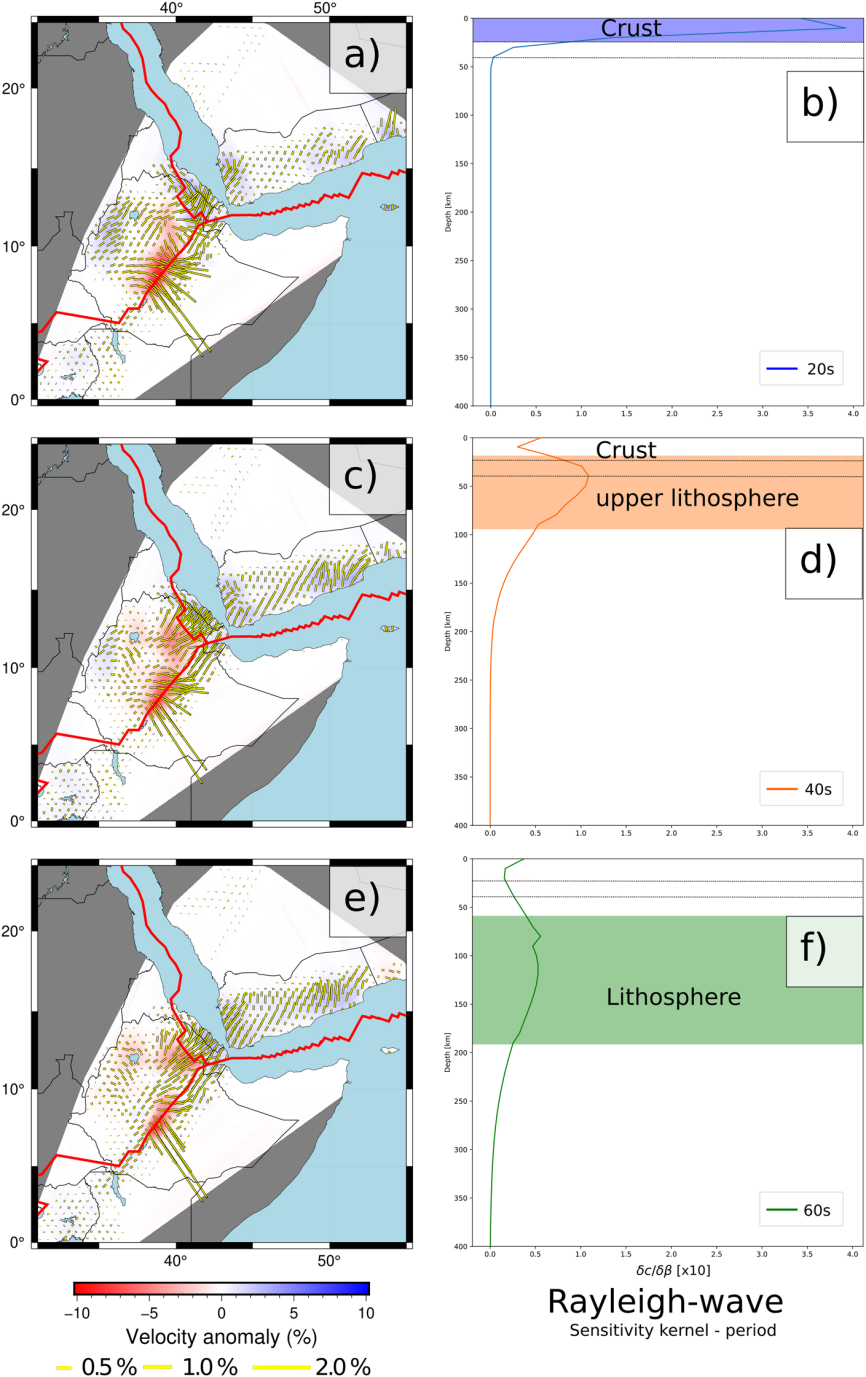
Isotropic and azimuthally anisotropic Rayleigh-wave phase velocity model at (**a**) 20s, (**c**) 40s and (**e**) 60s and corresponding sensitivity kernels (**b**, **d**, **f**). The colored rectangles indicate the regions with maximum sensitivity at each period respectively. Individual figure panels were generated with the Generic Mapping Tool (6.3.0)^51^ and PyGMT (0.7.0)^52^. Individual figure panels were combined using Inkscape (1.2.1)^53^.

Further, the dispersion curves were inverted for isotropic and azimuthally anisotropic Rayleigh-wave phase velocities^28^ at periods sampling the crust and lithospheric mantle, as displayed in Fig. 4. Rejection of outliers (Fig. S4) and inversion parameters (Figs. S5, S6, S7 and S8) have been determined using trade-off curves (Fig. S9). Careful examination of the path density (Fig. S10) and azimuthal coverage (Fig. S11), supplemented by resolution tests (Figs. S12 and S13), indicate that in the region around the Afar triple junction, the data coverage is sufficient to allow for the retrieval of both isotropic and anisotropic components. However, in the regions further away from the rift system, the station coverage does not allow for sufficient retrieval of both velocities and anisotropy. We will therefore focus our discussion in the region where the model appears to be well constrained.

## Results

The final models (Figs. 4, S14 and S15) and respective sensitivity kernels (Fig. S16) allowed us to investigate specific periods corresponding to crustal (10–30 s), intermediate (40–50 s), and lithospheric mantle (60 s and longer) depths. Representative periods have been selected to highlight our results (20, 40 & 60 s respectively in Fig. 4). Note that the sensitivity kernels have been computed using PREM^13^ as an initial model. These models display both lateral and vertical variations in Rayleigh-wave phase-velocity as well as azimuthal anisotropy.

In the period range sampling the crust (10–30 s), fast velocities are found outside the rift (up to +3%), associated with rift-parallel anisotropy, whereas rift-normal anisotropy and very slow velocities ($-$10%) are found at the center of the rift.

Then, at periods of 40–50 s, sampling both the crust and upper lithospheric mantle, the isotropic pattern remains very similar to that of shorter periods. The model is dominated by a strong negative velocity anomaly ($-$10%) in the backbone of the rift, and a gradual increase in velocities when moving away from the central part of the rift system. At those periods, the anisotropic pattern is also dominated by strong amplitudes in the central part of the rift (around 4%) with a rift-normal fast direction. In the flanks of the rift, the anisotropic pattern evolves with increasing periods, from rift-parallel directions (with amplitudes of 1%) in the northern flank at short periods (10–20 s) to rift-parallel (with amplitudes of 0.5%) at longer periods (40–50 s), consistent with the GPS^23^ and SKS^29,30^ measurements.

At periods of 60 s and longer, sampling the lithospheric mantle, very slow velocities ($-$10%) are still present in the central part of the rift, with the rift-normal anisotropy. In the northern vicinity of the rift, fast velocities are found (up to +4%), with rift-parallel fast direction of anisotropy with strong amplitudes (around 4%). Further to the north-west, low velocities ($-$3%) are found, with rift-normal anisotropy.

Beneath Yemen, where the resolution tests suggests that both isotropic and anisotropic pattern can be properly resolved (Figs. S12 and S13). Our results indicate a limited negative velocity anomaly at periods of 10 s ($-$1%) and a uniform EW fast direction of anisotropy (Figs. 4, S14 and S15. At periods of 20–80 s, fairly constant fast velocities (+2%) and a constant NE-SW fast direction of anisotropy are found while at periods of 100 s, an EW fast direction of anisotropy is found beneath Yemen: in its western part, fast velocities (+2%) and NE-SW fast direction of anisotropy are found, while in its eastern part, slow velocities ($-$1%) and WNW-ESE fast direction of anisotropy are found.

## Discussion

The isotropic pattern (Figs. 4, S14 and S15) is relatively monotonous at all period ranges. Our model is dominated by a very strong low-velocity signature beneath the East African Rift System, especially beneath the locations of active volcanoes (Fig. 1a). This is highly consistent with previous tomographic studies that found very slow velocities beneath the center of the Ethiopian rift in the crust^10–12^ and mantle^4–6^.

It is important to note that we are only looking at a 2D view of a complex 3D anisotropic system projected on horizontal maps. The very strong amplitudes of the Rayleigh-wave azimuthal anisotropy can also be linked with the vertically ascending mantle flow and the large volume of melt, which is also consistent with the “Null” measurements^31^ observed in the shear-wave splitting of the SKS phases in the region^7^. Recent studies focusing on shear-wave splitting measurements^7^ found that stations with null measurements were mainly located near Holocene volcanoes (Fig. 1a, suggesting the presence of fluid, melt, or ascending flow beneath the rift branches. This study also reveals rift-normal anisotropy beneath some stations in the northern flank of the rift and “Null” measurements of shear-wave splitting in the SW end of our study area^7^.

In addition, previous studies focusing on shear-wave splitting measurements^7,21,22^ and the surface velocity vectors^23,32^ display a homogeneous pattern aligned with the direction of the Ethiopian Rift System (Fig. 1). This very straightforward pattern displayed by both shear-wave splitting and geodetic measurements is highly contrasting with the complexity of the crustal system beneath the region. The crustal material in the region is known to be highly heterogeneous. It involves a complex system composed of vertical layering of magmatic reservoirs in both the upper and lower crust^33^. Moreover, current volcanic activity is strongly affecting the present state of the crust near the rift branches^12,34,35^.

The NE-SW directions observed by GPS and SKS studies agree well with the overall direction of the Arabian Plate^23,36^, and the direction of the opening of the Ethiopian rift^32,37^. The plate motion of the Somalian Plate (EW), the direction of opening of the Gulf of Aden (EW) and the Red Sea Rift (NNW-SSE) are well determined by GPS velocity vectors. Further from the rift system, limited information on the shear-wave splitting is available from the global database^29,30^. The overall pattern of anisotropy measured by shear-wave splitting and the GPS velocity field in the region is overall consistent with global tomographic studies^38,39^, where similar trends of anisotropy are found at lithospheric and asthenospheric depths (in the range 80–330 km).

The fast direction of anisotropy (Figs. 4, S14 and S15), however, displays two distinct patterns: one in the period range of 10–30 s and another one in the period range of 60–100 s. At periods sampling the crust (10–30 s), rift-normal anisotropy is found beneath the center of the rift, but in its flank, mostly rift-parallel anisotropy is found. At periods sampling the lithosphere (60–100 s), rift-normal anisotropy is still present beneath the center of the rift, whereas a band of 200–300 km width around both sides of the rift exhibits rift-parallel anisotropy. Beneath the Nubia Plate, in the northern part of the rift, the anisotropic pattern exhibits mostly a rift-parallel direction of fast propagation in the period range 10–30 s, which is consistent with the shear-wave splitting measurements^29,30^. In contrast, the rift-normal directions of fast propagation are found in the period range 60–100 s.

In the literature, conflicting models have been proposed to explain the distribution of the anisotropy in the eastern Horn of Africa. Some suggested a deep origin of the anisotropy, with a source located around 300 km depth in the asthenospheric mantle^22^. In contrast, other studies suggested that the anisotropy originates from crustal and lithospheric deformation^40,41^. In our models, the distribution of the fast directions of anisotropies is consistent with the overall pattern of anisotropy obtained by shear-wave splitting measurements^29,30^. Outside the branches of the rift, the SKS measurements agree with the directions of anisotropies found at shorter periods sampling the crust. These directions of anisotropies are very different in the lithospheric mantle, suggesting a potential decoupling between the heterogeneous crust and the lithospheric mantle beneath the East African Rift System. This also suggests that the shear-wave splitting measurements are primarily sensitive to crustal materials in this region. Beneath the Nubia Plate, in the northern part of the rift, the seismic anisotropy observed at crustal depths (10–30 s), is perpendicular to the seismic anisotropy observed at mantle depths (60–100 s). This suggests a decoupling between the crust and mantle in the region. Additionally, this raises the possibility of a weak lithospheric mantle, which may be mechanically or thermally weakened by the opening of the rift branches on its northern and western flanks and/or by the rift’s vicinity.

Geodynamic models of the region^3,42^ have already predicted the decoupling between the crust and mantle in the Afar region. The suggested initially weakened rigid lithosphere lid is affected by multi-directional, asymmetric far-field stresses. These geodynamic models agree well with our results.

In the center of the rift, we found a very strong anisotropy, with amplitudes of up to 4%, contrasting with the surroundings, where amplitudes are in the order of 0.5–1%. This is also in good agreement with previous reports of shear-wave splitting measurements^29,30^ that found the delay time of 1.5 s near the location of Holocene volcanoes^43^. This very strong anisotropy is considered to originate from the combined effects of heterogeneous crustal material^33^, the high temperature^14^, the presence of partial melt^15^, and ascending mantle flow^44^.

## Conclusion

Our results provide additional constraints on isotropic and anisotropic patterns in the eastern part of the Horn of Africa. Our tomographic model highlight sharp structural contrasts between the active rift system, depicted by the presence of Holocene volcanoes at the surface and display outstandingly negative velocity anomalies ($-$10%) as well as significantly high anisotropy (> 4%). By contrast, the surrounding of the rift system exhibit relatively positive velocity anomalies (+4%) and little anisotropy (in the order of 1%), suggesting a mechanical decoupling between the crust and lithospheric mantle in this region.

## Methods

In order to build Rayleigh-wave phase velocity anisotropic maps beneath the Afar region, we followed a three-step method. The first step consists in collecting and pre-processing seismic data from regional and teleseismic earthquakes. The second step involves the computation of a set of phase-velocity dispersion curves between pairs of stations. And in the last step, those dispersion curves are inverted for isotropic and azimuthally anisotropic phase-velocity maps at selected periods^45,46^.

Our database encompasses the records of 4137 selected regional and teleseismic earthquakes recorded by 361 seismic stations (Fig. 2). A total of 68,420 dispersion curves were measured to constrain 3578 path-average dispersion curves for interstation distances in the range of 30–2150 km. This set of dispersion curve is then inverted for both isotropic and azimuthally anisotropic Rayleigh-wave phase velocity model at periods sampling the crust and lithospheric mantle. The inversion is based on an LSQR approach^47,48^, and the average of all dispersion curves in the region is used as an initial model.

At each point of the model, the total velocity anomaly can be parameterized with five coefficients: one for the isotropic phase-velocity variation, $\delta C_{iso}$, two for the 2$\psi$-anomaly, $A_{2\psi }$ and $B_{2\psi }$, and two for the 4$\psi$-anomaly, $A_{4\psi }$ and $B_{4\psi }$:

1 $$\begin{aligned} \delta C = \delta C_{iso} + A_{2\psi } cos(2\psi ) + B_{2\psi } sin(2\psi ) + A_{4\psi } cos(4\psi ) + B_{4\psi } sin(4\psi ) . \end{aligned}$$

We used a triangular grid of knots^27^, with a grid spacing of 10 km. The data coverage is not constant at all periods, resulting in some differences in the grids, composed of 926 knots at 20 s, 924 knots at 40 s, and 903 knots at 60 s. Each dispersion curve yields the average phase velocity along the path linking the two stations as a function of period, and the total average velocity anomaly along this path **is** written as the integral of local anomalies at each grid knot sampled by the given path,

2 $$\begin{aligned} \delta \bar{C_{i}} = \int _{\varphi }\int _{\theta } K_{i}(\varphi ,\theta ) \; \delta C(\varphi ,\theta ) \; d\theta \; d\varphi \; , \end{aligned}$$

where the local anomalies $\delta C(\varphi ,\theta )$ are weighted with respect to the sensitivity kernels $K_{i}(\varphi ,\theta )$. The sensitivity kernel represents the weighted contribution of a specific path to the total velocity anomaly at each knot.

## Supplementary Information

Supplementary Information.

## Data Availability

Dispersion curves and tomographic models are available at https://github.com/cplegendre/Afar-2sta.

## Code availability

The codes used to compute the tomographic model and all derived results are available from the corresponding author upon reasonable request.
