# Supplementary material for: Crust-mantle decoupling beneath Afar revealed by Rayleigh-wave tomography
Source: Sci Rep. 2022 Oct 11;12:17036. doi: 10.1038/s41598-022-20890-5 (PMC9553937; doi:10.1038/s41598-022-20890-5)
Supplement: Supplementary file 1 — Supplementary Information. [file 41598_2022_20890_MOESM1_ESM.pdf]

Supplementary information for:
Crust-mantle decoupling beneath Afar revealed by Rayleigh-wave tomography

Utpal Kumar[1] and Cédric P. Legendre[2,*]

[1] *Berkeley Seismological Laboratory, University of California, Berkeley, CA, USA.*

[2] *Institute of Geophysics, Czech Academy of Science, Bočni II, 1401, 141 31 Prague, Czech Republic*

[*] *legendre@ig.cas.cz*

Abstract

The Afar triple junction accustoms the diverging plate dynamics between the Arabian, Nubian, and Somalian plates along the Red Sea, Gulf of Aden, and East African rifts. The average anisotropy obtained from shear-wave splitting measurements is in good agreement with the surface motion recovered by geodetic analyses. Here, we use seismic time-series to map Rayleigh-wave azimuthal anisotropy in the crust and lithospheric mantle beneath the East African Rift System. Our results suggest that a layering of anisotropy is present around the East African Rift System. At shorter periods sampling the crust, rift parallel anisotropy is present in the vicinity of the rift, but in the central part of the rift, rift normal anisotropy is found. At longer periods, sampling the lithospheric mantle, the opposite anisotropic pattern is observed. These observations suggest that the crust and lithospheric mantle are mechanically decoupled beneath the environs of the East African Rift System, as well as complex dynamics within the crust and lithosphere.

List of Figures

S1 Seismicity . . . 3
S2 Event selection . . . 4
S3 Dispersion curves . . . 5
S4 Outliers rejection . . . 6
S5 Isotropic smoothing . . . 7
S6 Anisotropic smoothing . . . 8
S7 Isotropic damping . . . 9
S8 Anisotropic damping . . . 10
S9 Trade-off curves . . . 11
S10 Path density . . . 12
S11 Path coverage . . . 13
S12 Resolution tests - 1 . . . 14
S13 Resolution tests - 2 . . . 15
S14 Models . . . 16
S15 Zoom-in models . . . 17
S16 Sensitivity kernels . . . 18

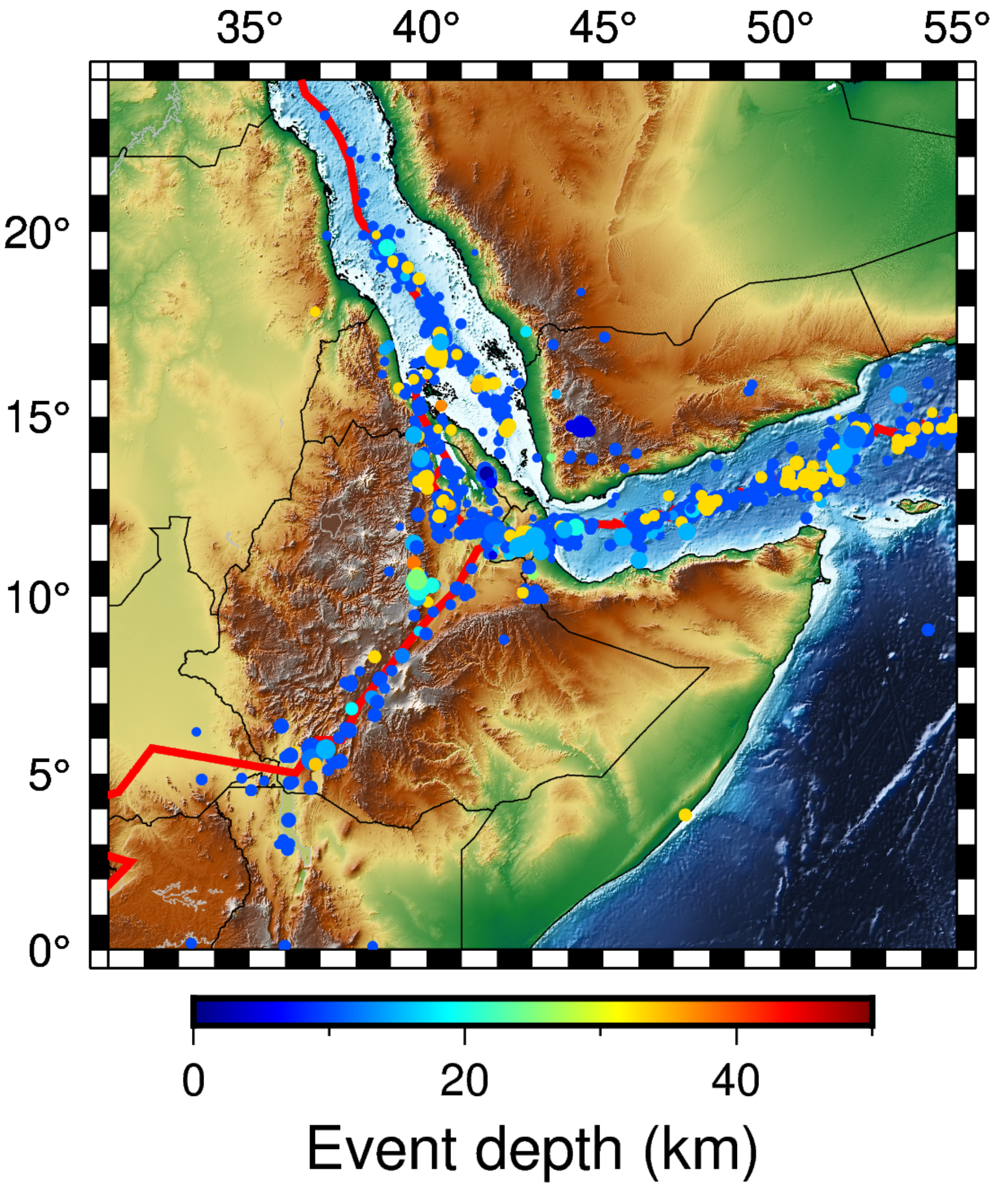

Figure S1: Regional tectonic map of Afar region. Plate boundaries [1] are framed in red. Events ($M_w > 4.0$) occurring between 1950 and 2021 reported in the region. Earthquake depth is indicated by the color scale, and the size of the circle is representative of its magnitude ($4.0 > M_w > 6.6$). Figure was generated with the Generic Mapping Tool (6.3.0) [2] and PyGMT (0.7.0) [3].

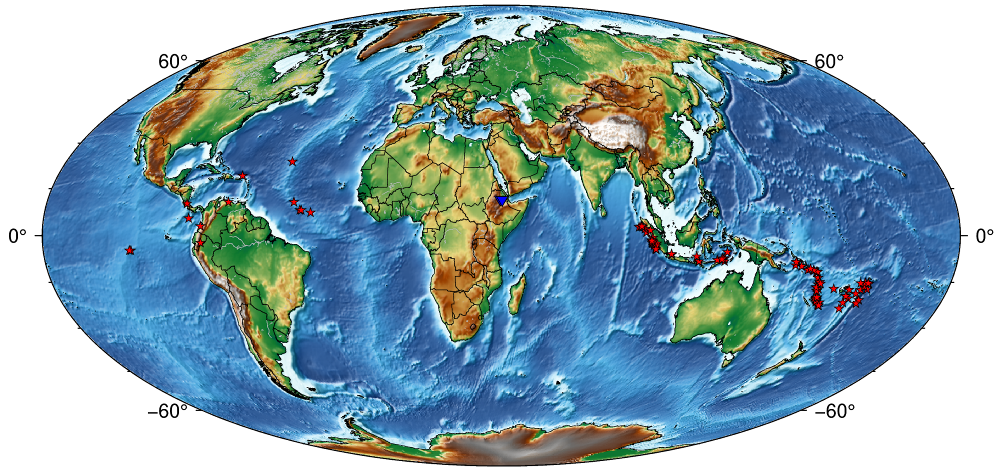

Figure S2: Selected events for the station pair CHIE-MILE. Figure was generated with the Generic Mapping Tool (6.3.0) [2] and PyGMT (0.7.0) [3].

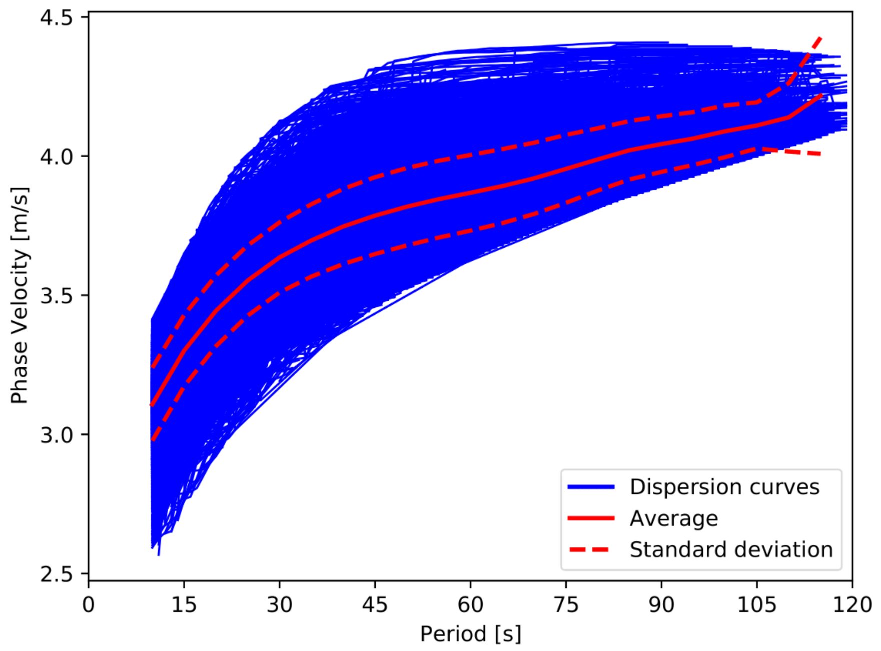

Figure S3: Automatically measured phase-velocity curves (blue) for all station pairs. Average of all the dispersion curves (red solid line) and standard deviation (red dashed lines).

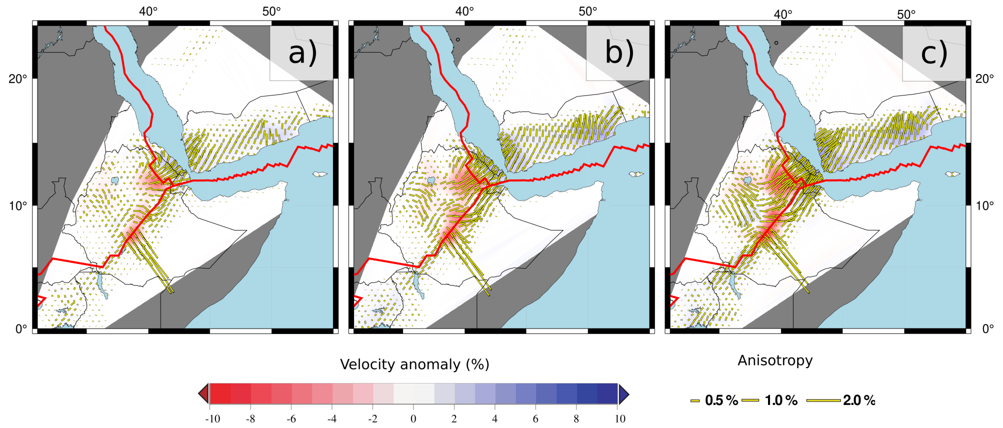

Figure S4: Effect of data rejection with 50% (a), 80% (b) and 100% (c) of the dispersion kept. Individual figure panels were generated with the Generic Mapping Tool (6.3.0) [2] and PyGMT (0.7.0) [3]. Individual figure panels were combined using Inkscape (1.2.1) [4].

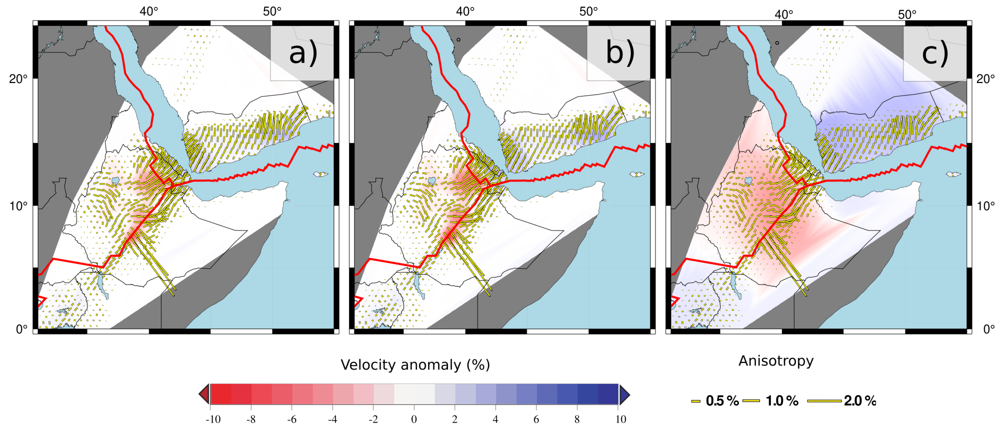

Figure S5: Effect of isotropic smoothing: Low (a), preferred (b) and high (c) smoothing of the isotropic component. Individual figure panels were generated with the Generic Mapping Tool (6.3.0) [2] and PyGMT (0.7.0) [3]. Individual figure panels were combined using Inkscape (1.2.1) [4].

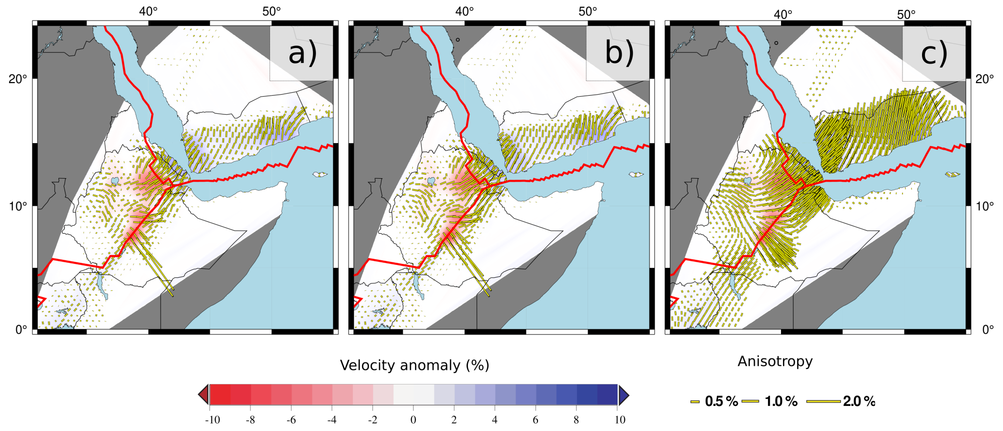

Figure S6: Effect of anisotropic smoothing: Low (a), preferred (b) and high (c) smoothing of the anisotropic component. Individual figure panels were generated with the Generic Mapping Tool (6.3.0) [2] and PyGMT (0.7.0) [3]. Individual figure panels were combined using Inkscape (1.2.1) [4].

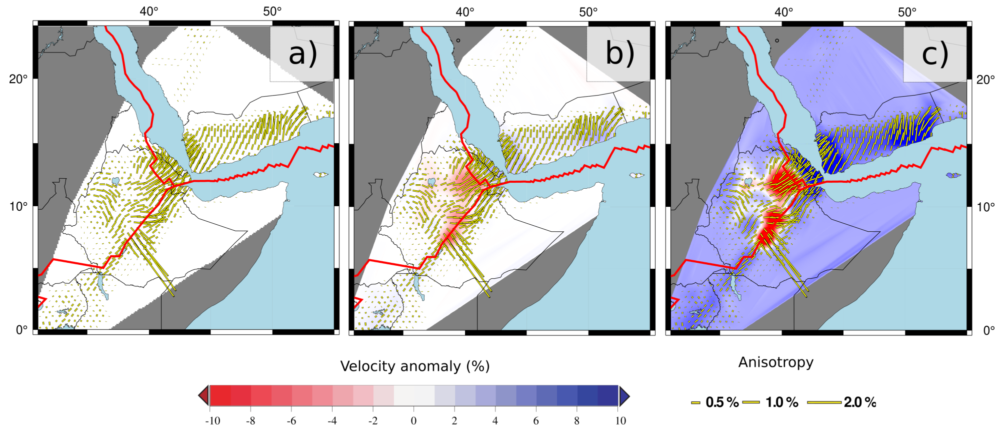

Figure S7: Effect of isotropic damping: Low (a), preferred (b) and high (c) damping of the isotropic component. Individual figure panels were generated with the Generic Mapping Tool (6.3.0) [2] and PyGMT (0.7.0) [3]. Individual figure panels were combined using Inkscape (1.2.1) [4].

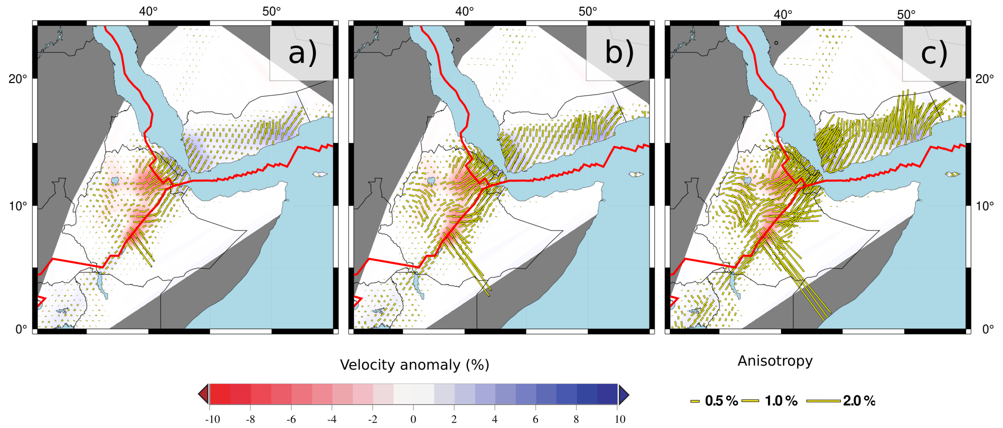

Figure S8: Effect of anisotropic damping: Low (a), preferred (b) and high (c) damping of the anisotropic component. Individual figure panels were generated with the Generic Mapping Tool (6.3.0) [2] and PyGMT (0.7.0) [3]. Individual figure panels were combined using Inkscape (1.2.1) [4].

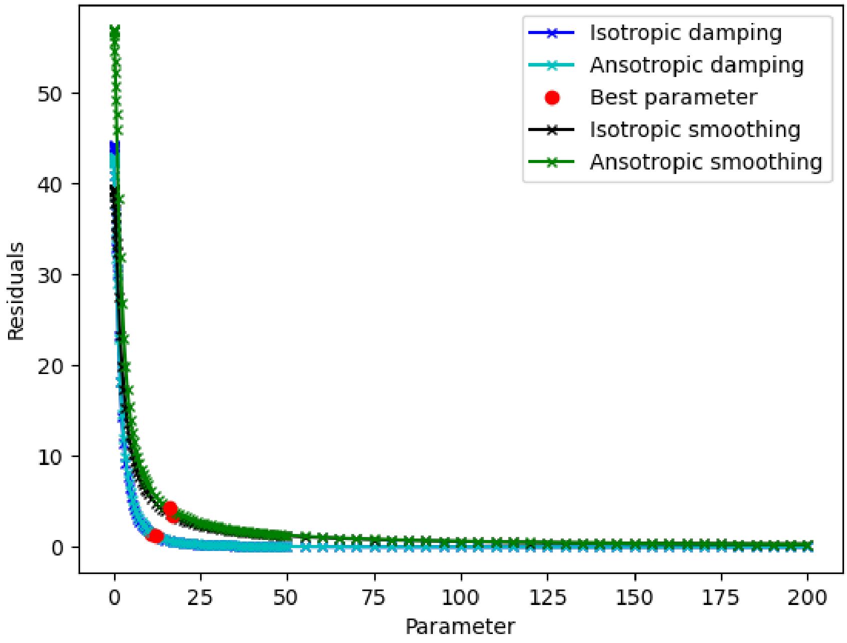

Figure S9: L-curves used to determine the optimal parameter for the isotropic and anisotropic smoothing and damping.

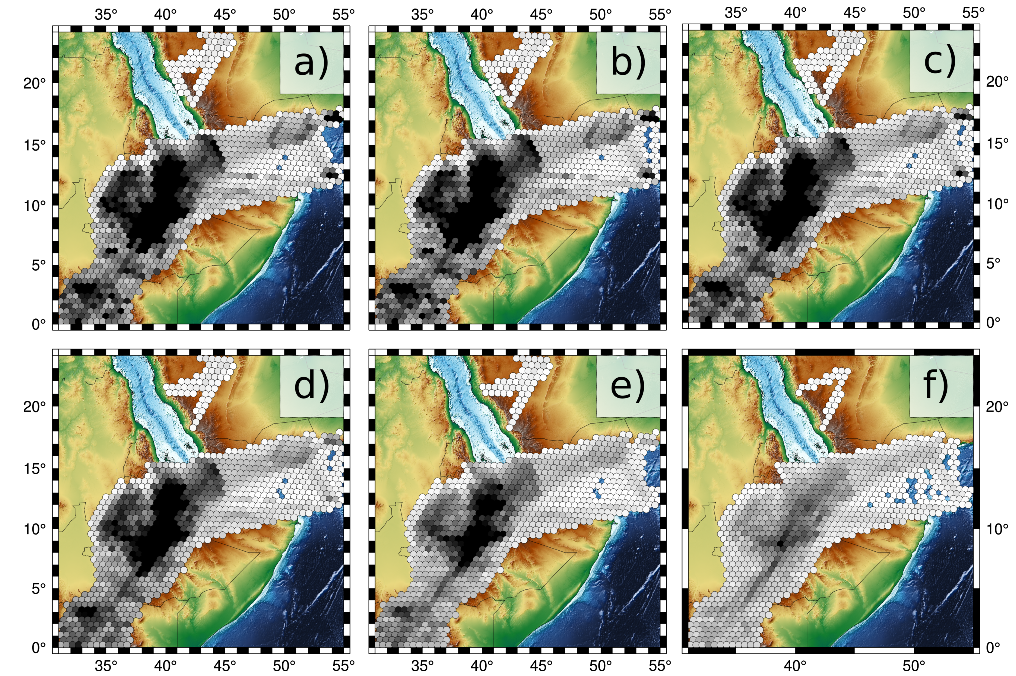

Figure S10: Path density in the region indicated with white-to-black scale at periods of 10 s (a), 20 s (b), 40 s (c), 60 s (d), 80 s (e), 100 s (f). Individual figure panels were generated with the Generic Mapping Tool (6.3.0) [2] and PyGMT (0.7.0) [3]. Individual figure panels were combined using Inkscape (1.2.1) [4].

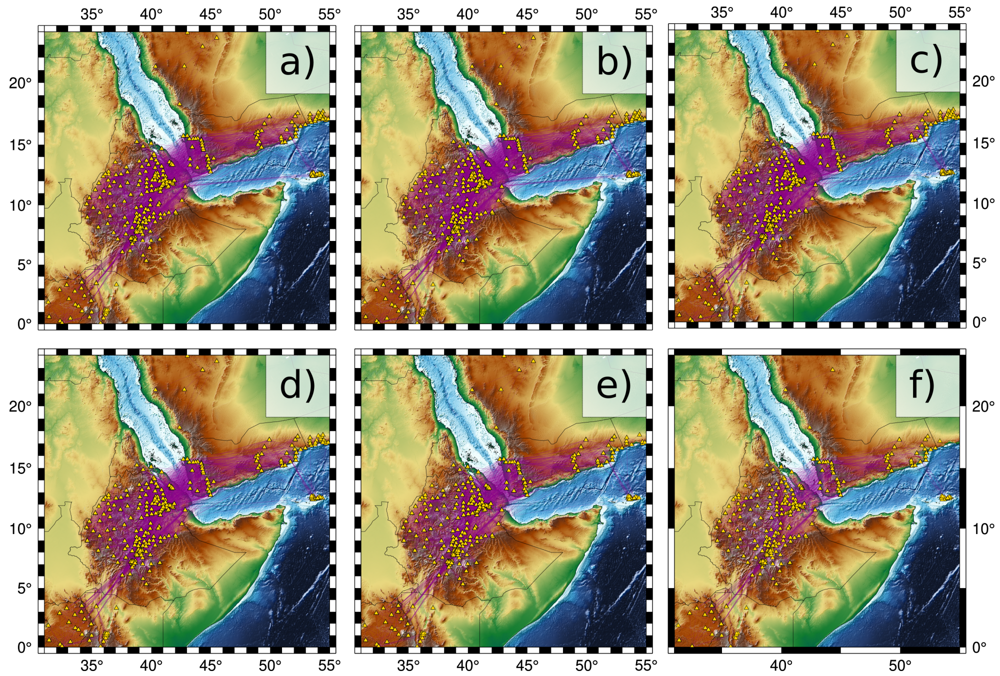

Figure S11: Inter-station paths in the region indicated with seismic stations (blue triangles) linked by a brown line at periods of 10 s (a), 20 s (b), 40 s (c), 60 s (d), 80 s (e), 100 s (f). Individual figure panels were generated with the Generic Mapping Tool (6.3.0) [2] and PyGMT (0.7.0) [3]. Individual figure panels were combined using Inkscape (1.2.1) [4].

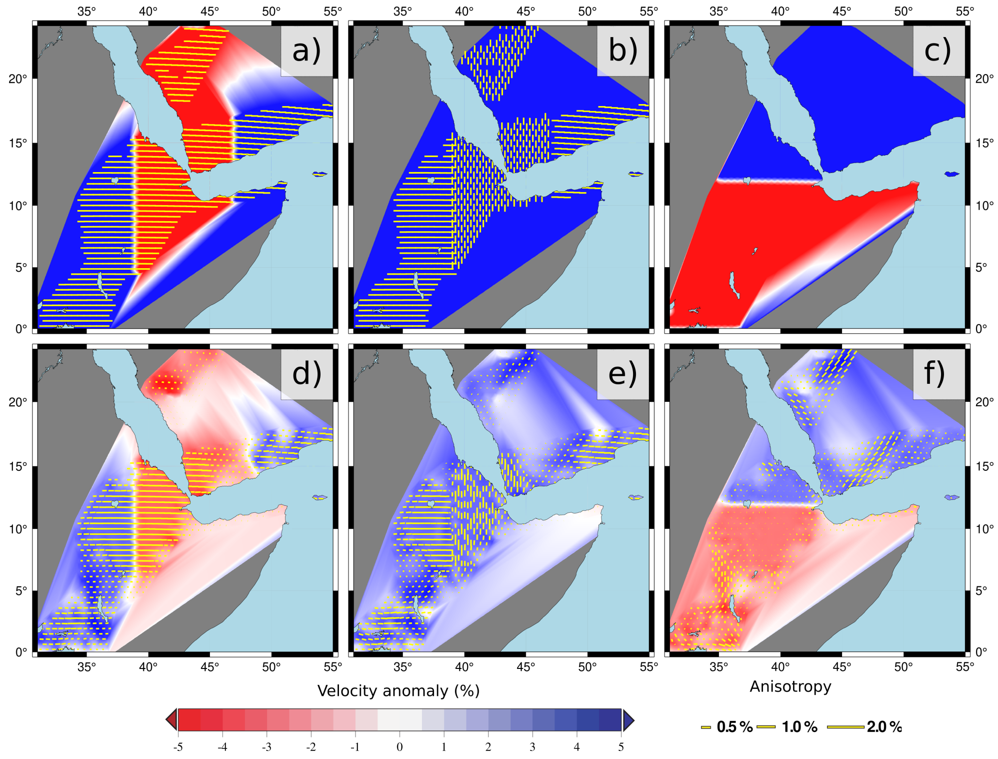

Figure S12: Input test models at periods of 10, 20, 40 s (a-c) and reconstructed models for respective periods (d-f). Individual figure panels were generated with the Generic Mapping Tool (6.3.0) [2] and PyGMT (0.7.0) [3]. Individual figure panels were combined using Inkscape (1.2.1) [4].

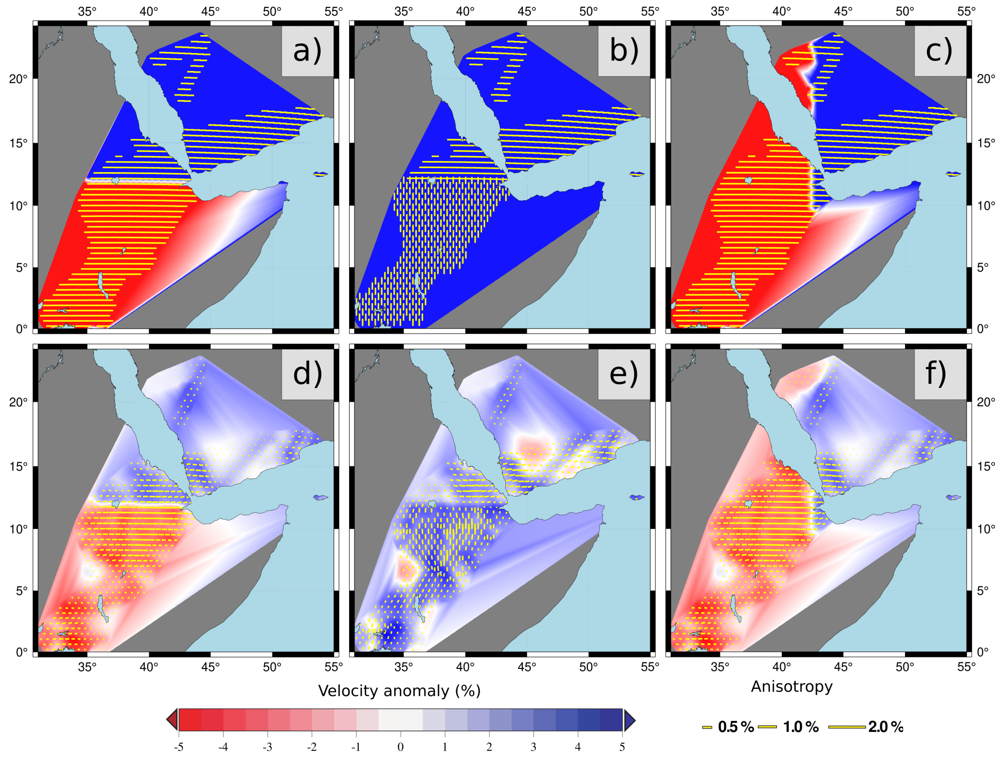

Figure S13: Input test models at periods of 60, 80, 100 s (a-c) and reconstructed models for respective periods (d-f). Individual figure panels were generated with the Generic Mapping Tool (6.3.0) [2] and PyGMT (0.7.0) [3]. Individual figure panels were combined using Inkscape (1.2.1) [4].

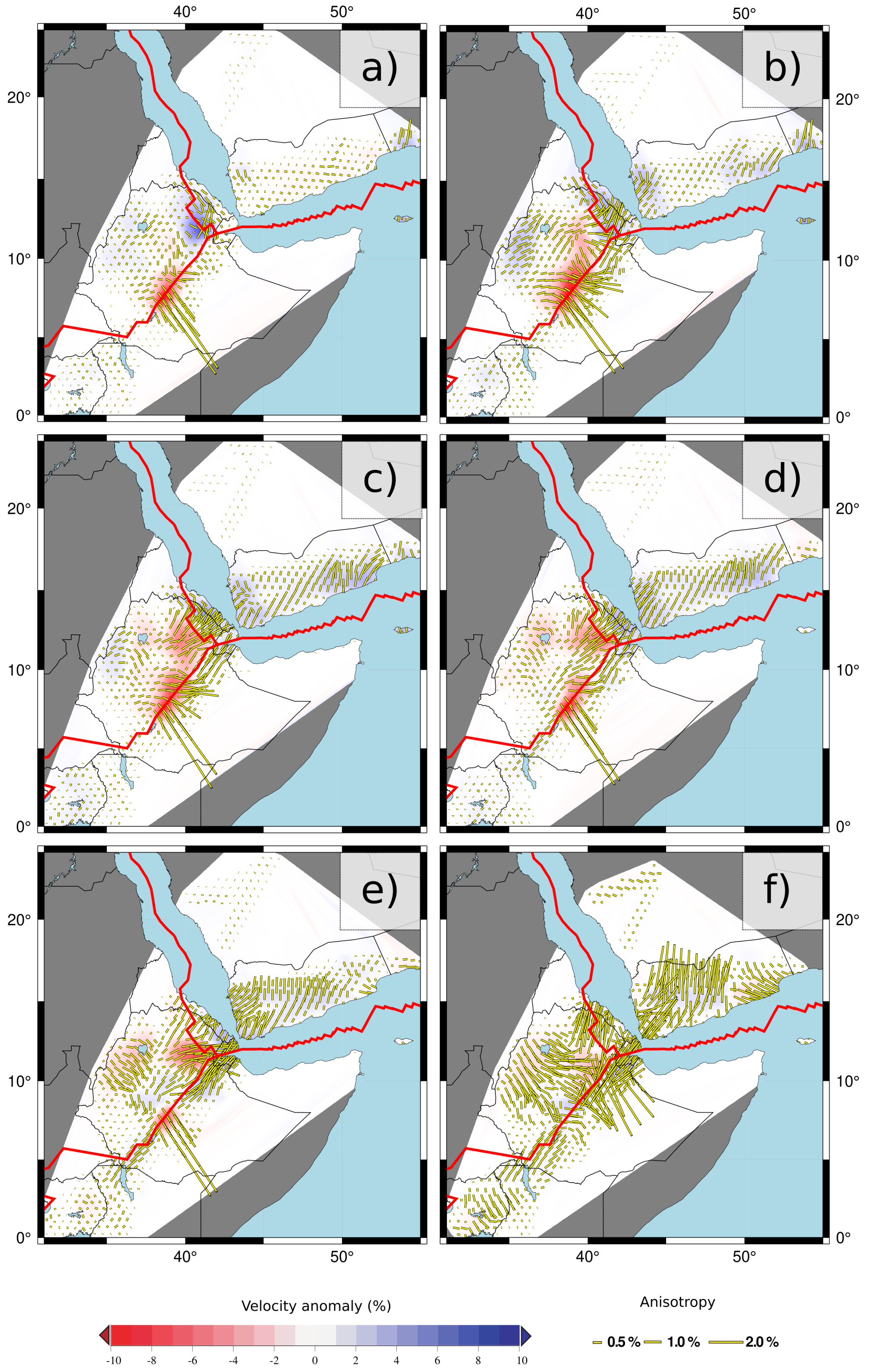

Figure S14: Isotropic and azimuthally anisotropic Rayleigh-wave phase velocity model periods of 10 s (a), 20 s (b), 40 s (c), 60 s (d), 80 s (e), 100 s (f). Individual figure panels were generated with the Generic Mapping Tool (6.3.0) [2] and PyGMT (0.7.0) [3]. Individual figure panels were combined using Inkscape (1.2.1) [4].

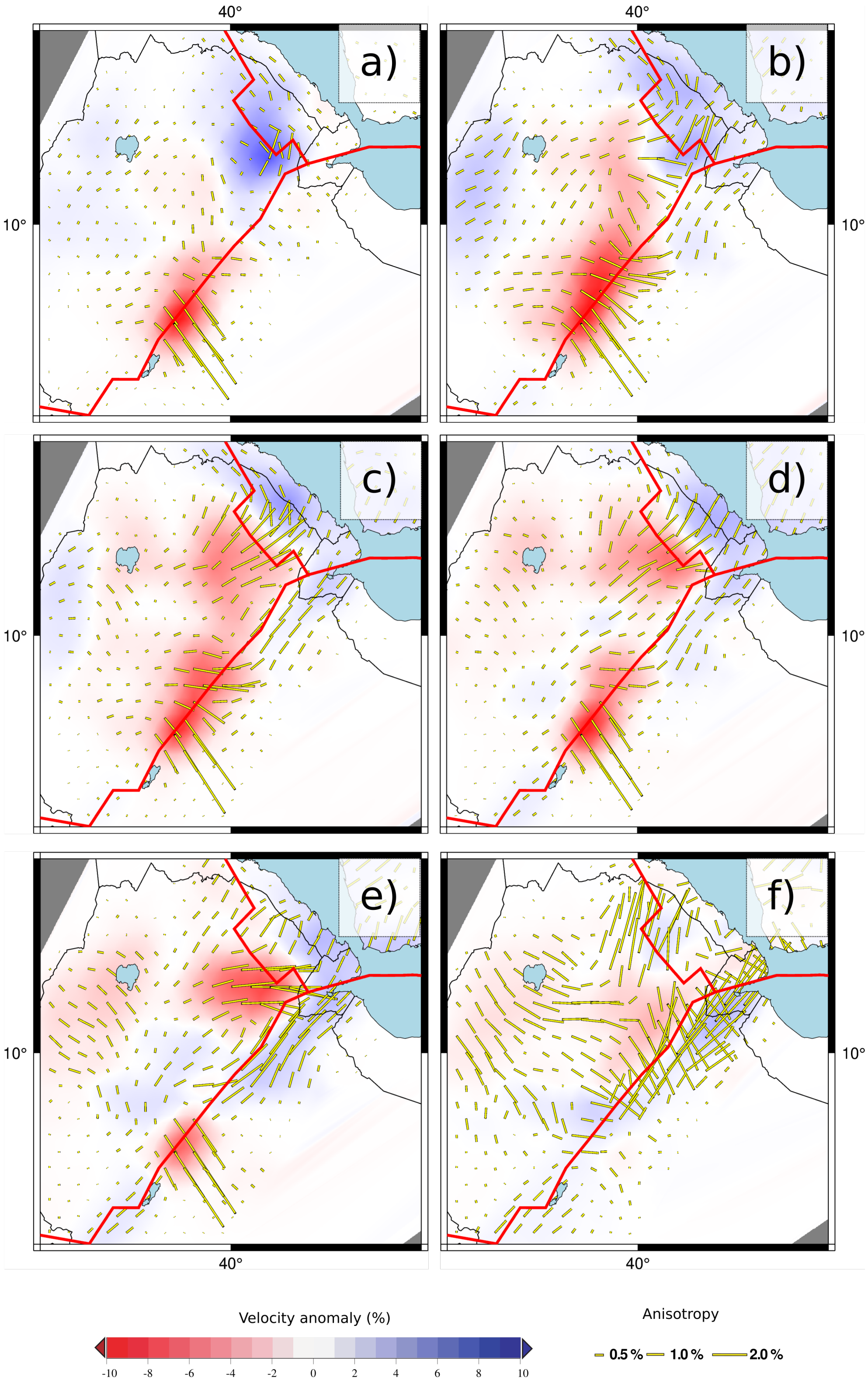

Figure S15: Zoom in of the isotropic and azimuthally anisotropic Rayleigh-wave phase velocity model periods of 10 s (a), 20 s (b), 40 s (c), 60 s (d), 80 s (e), 100 s (f). Individual figure panels were generated with the Generic Mapping Tool (6.3.0) [2] and PyGMT (0.7.0) [3]. Individual figure panels were combined using Inkscape (1.2.1) [4].

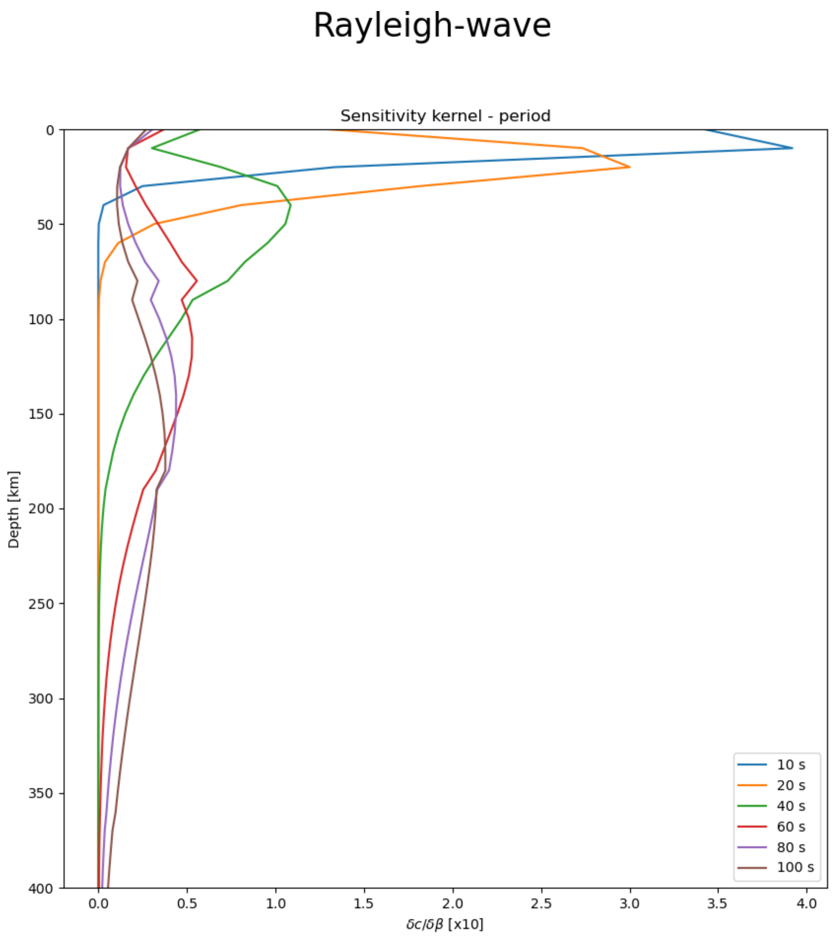

Figure S16: Sensitivities of the Rayleigh-wave phase velocities to shear-wave speed as functions of depth for selected periods.

References

[1] Bird, P.: An updated digital model of plate boundaries. Geochemistry, Geophysics, Geosystems **4**, 3–1027 (2003)

[2] Wessel, P., Luis, J., Uieda, L., Scharroo, R., Wobbe, F., Smith, W., Tian, D.: The generic mapping tools version 6 (6.3.0). Geochemistry, Geophysics, Geosystems **20**(11), 5556–5564 (2019)

[3] Uieda, L., Tian, D., Leong, W.J., Jones, M., Schlitzer, W., Grund, M., Toney, L., Yao, J., Magen, Y., Materna, K., Newton, T., Anant, A., Ziebarth, M., Quinn, J., Wessel, P.: PyGMT (0.7.0): A Python Interface for the Generic Mapping Tools. `https://doi.org/10.5281/zenodo.6702566`. `https://doi.org/10.5281/zenodo.6702566`

[4] Inkscape Project: Inkscape (1.2.1) [Internet] (2022). `https://inkscape.org`
